# Thixotropic Red Microalgae Sulfated Polysaccharide-Peptide Composite Hydrogels as Scaffolds for Tissue Engineering

**DOI:** 10.3390/biomedicines10061388

**Published:** 2022-06-11

**Authors:** Michal Halperin-Sternfeld, Gal Netanel Liberman, Raha Kannan, Francesca Netti, Peter X. Ma, Shoshana Malis Arad, Lihi Adler-Abramovich

**Affiliations:** 1 Department of Oral Biology, The Maurice and Gabriela Goldschleger School of Dental Medicine, Sackler Faculty of Medicine, Tel Aviv University, Tel Aviv 6997801, Israel; michal4@mail.tau.ac.il (M.H.-S.); francescanetti1990@gmail.com (F.N.); 2 The Center for Nanoscience and Nanotechnology, Tel Aviv University, Tel Aviv 6997801, Israel; 3 The Center for the Physics and Chemistry of Living Systems, Tel Aviv University, Tel Aviv 6997801, Israel; 4 Avram and Stella Goldstein-Goren Department of Biotechnology Engineering, Ben-Gurion University of the Negev, Beer-Sheva 84105, Israel; galnet@post.bgu.ac.il (G.N.L.); arad@post.bgu.ac.il (S.M.A.); 5 Department of Biologic and Materials Sciences, University of Michigan, Ann Arbor, MI 48109, USA; rakannan@umich.edu (R.K.); mapx@umich.edu (P.X.M.)

**Keywords:** sulfated polysaccharides, tissue engineering, biomaterials, hydrogels

## Abstract

Sulfated polysaccharides of red marine microalgae have recently gained much attention for biomedical applications due to their anti-inflammatory and antioxidant properties. However, their low mechanical properties limit their use in tissue engineering. Herein, to enhance the mechanical properties of the sulfated polysaccharide produced by the red marine microalga, *Porphyridium* sp. (PS)*,* it was integrated with the fluorenylmethoxycarbonyl diphenylalanine (FmocFF) peptide hydrogelator. Transparent, stable hydrogels were formed when mixing the two components at a 1:1 ratio in three different concentrations. Electron microscopy showed that all hydrogels exhibited a nanofibrous structure, mimicking the extracellular matrix. Furthermore, the hydrogels were injectable, and tunable mechanical properties were obtained by changing the hydrogel concentration. The composite hydrogels allowed the sustained release of curcumin which was controlled by the change in the hydrogel concentration. Finally, the hydrogels supported MC3T3-E1 preosteoblasts viability and calcium deposition. The synergy between the sulfated polysaccharide, with its unique bioactivities, and FmocFF peptide, with its structural and mechanical properties, bears a promising potential for developing novel tunable scaffolds for tissue engineering that may allow cell differentiation into various lineages.

## 1. Introduction

Tissue engineering primarily aims at generating artificial constructs to replace and restore dysfunctional tissues [1]. Traditionally, scaffolds for tissue engineering have been combined with cells and growth factors to enhance their bioactivity [1]. However, cell- and growth factor-free scaffolds have recently emerged as a new tissue engineering paradigm. They overcome limitations associated with cells and growth factors-containing scaffolds, including decreased cell survival, immunogenic potential [2], and loss of growth factor bioactivity [3]. Cell-free scaffolds are designed to mimic the natural extracellular matrix (ECM) to effectively recruit the host’s native cells while providing provisional structural support for the recruited cells to promote in situ tissue regeneration.

Natural polysaccharides have emerged as promising candidates to serve as natural products to be applied in tissue engineering [4,5,6]. Natural polysaccharides comprise essential components present in the native ECM and exhibit biocompatibility, biodegradability, and recognition sites for cell attachment [7]. In particular, sulfated polysaccharides are widely known for their ability to bind several cell receptors involved in cell adhesion, proliferation and differentiation [8]. Sulfated polysaccharides isolated from marine algae hold pharmacological attributes that vary among the different species [8,9,10]. Specifically, we have shown that a sulfated polysaccharide produced from red microalgae possesses various bioactivities, including anti-inflammatory, antioxidant, and antiviral functions [11]. This polysaccharide is derived from the red marine microalga *Porphyridium* sp. and has a high molecular weight of 3000–5000 kDa with a negative charge due to half-ester sulfate groups and glucuronic acid [12]. Furthermore, it is resistant to hyaluronidase and stable at a wide range of temperatures and pH levels [13]. The polysaccharide has been studied as a lubricating agent [14] for its potential in pharmaceutics, food and drug delivery [15], and as a potential for wound dressing material when combined with chitosan and zinc ions [11,16,17]. Despite the significant advantages, polysaccharide shows inadequate mechanical properties and fast degradation rates [18,19], similar to other polysaccharides, limiting its use in tissue engineering.

Polysaccharides in general and polysaccharides from *Porphyridium* sp. are often modified and strengthened by cross-linking, chemical coating, and the addition of synthetic materials. However, their degradation may result in the release of toxic products [20]. Short aromatic peptides, particularly fluorenylmethoxycarbonyl-diphenylalanine (FmocFF), have been recently proposed to reinforce natural polysaccharides to produce scaffolds for tissue culture, bone regeneration and sustained release of bioactive molecules [21,22,23]. Unlike conventional polymeric hydrogels based on a covalently cross-linked polymer network, short aromatic peptides can form self-supporting nanostructured supramolecular hydrogels through noncovalent interactions of the molecules in a self-assembly process [24]. When combined with sodium alginate, hyaluronic acid, or konjac glucomannan, FmocFF peptide has been shown to increase their rigidity and stability, resulting in ECM-mimicking hydrogel biomaterials for potential use in tissue engineering and drug delivery [21,22,25,26].

Here, we integrated the polysaccharides of the red microalga *Porphyridium* sp. (PS) with FmocFF peptide nanostructures to form ECM-mimicking composite hydrogel scaffolds for tissue engineering, harnessing the similarity of FmocFF and the PS to the fibrous proteins and glycosaminoglycans in the ECM, respectively. We demonstrate the formation of biocompatible composite FmocFF/PS hydrogels with improved mechanical properties compared to polysaccharide alone. Tunable mechanical properties were obtained upon changing the concentration of the components. Furthermore, the formed hydrogels allowed MC3T3-E1 preosteoblasts proliferation, osteodifferentiation and biomineralization. Finally, the hydrogels served as vehicles for curcumin release. We believe that the synergy between the polysaccharide, with its unique structure and bioactivity, and FmocFF, with its structural and mechanical properties, bears a promising potential for developing novel scaffolds for three-dimensional cell culture and tissue engineering.

## 2. Materials and Methods

### 2.1. Materials

Lyophilized Fmoc-Phe-Phe-OH (FmocFF) peptide was purchased from Bachem (Budendorf, Switzerland) and the PS was produced as previously described [16,27]. The 3-[4,5-dimethylthiazole-2-yl]-2,5-diphenyltetrazolium bromide (MTT), Alizarin red, Cavacurcumin, and 9 mm diameter × 2 mm depth silicone isolators were purchased from Sigma Aldrich (Rehovot, Israel). Calcium Reagent Set was purchased from Pointe Scientific (Canton, MI, USA). MC3T3-E1 Subclone 4 (CRL-2593) preosteoblasts were purchased from ATCC (Manassas, VA, USA). Alpha Minimum Essential Medium (α-MEM), fetal bovine serum (FBS), fetal calf serum (FCS), penicillin and streptomycin were purchased from Biological Industries (Beit-Haemek, Israel).

### 2.2. Methods

#### 2.2.1. Preparation of FmocFF/PS Composite Hydrogels

FmocFF peptide was dissolved in dimethyl sulfoxide (DMSO) solvent to prepare a stock solution of 100 mg peptide in 1 mL of DMSO by vortexing until a transparent solution was obtained. The peptide stock solution was added to the PS (from a 1% stock), diluted in ddH_2_O and vortexed to form hydrogels at 1:1 ratio and final concentrations of 0.5% *w*/*w* (5 mg/mL), 1% *w*/*w* (10 mg/mL), and 2% *w*/*w* (20 mg/mL). 1 mL of 0.5% hydrogel was composed of 25 µL of FmocFF stock solution diluted in 250 mg PS in 727 µL ddH_2_O. The 1% hydrogel was composed of 50 µL of FmocFF stock solution diluted in 500 mg PS in 450 µL ddH_2_O. The 2% hydrogel was composed of 100 µL of FmocFF stock solution diluted in 900 mg PS without ddH_2_O.

#### 2.2.2. Transmission Electron Microscopy (TEM)

FmocFF/PS composite hydrogels (10 µL) were placed on a 400-mesh copper grid and excess fluid was removed. Negative staining was achieved by deposition of 10 µL of 2% uranyl acetate in water. After 2 min, excess uranyl acetate was removed. The sample was viewed using a JEM-1400Plus TEM (JEOL USA, MA), operating at 80 kV.

#### 2.2.3. Scanning Electron Microscopy (SEM)

The surface morphology of the hydrogels was examined using SEM. Hydrogel samples were attached to aluminum SEM specimen-mounting stubs and sputter-coated with a gold–palladium alloy using a Sputter Coater. The morphology of the hydrogels was observed by JSM-IT100 InTouchScope™ (JEOL USA, MA) operating at 20 kV.

#### 2.2.4. Rheological Measurements

Rheological analyses of the hydrogels were carried out using an AR-G2 rheometer (TA Instruments, New Castle, DA, USA) equipped with a 20 mm diameter parallel-plate geometry. Strain sweep experiments were performed from 0.1% to 100% strain and frequency sweep experiments between 0.01–100 Hz using 220 µL of a freshly prepared sample (resulting in a gap size of 0.6 mm) to determine the limit of the linear viscoelastic region (LVR). To evaluate the storage modulus (G’) and loss modulus (G”), time sweep experiments were recorded at 5 Hz oscillation and 0.1% strain deformation [28]. To test the thixotropy of the hydrogels, 100% and then 0.1% strains were applied at a constant frequency of 5 Hz [29]. The complex viscosities of hydrogels were measured versus the frequency.

#### 2.2.5. Fourier-Transform Infrared (FTIR) Spectroscopy

FTIR spectra were collected using a Thermo Nicolet Nexus 470 FTIR spectrometer (GMI, MN, USA) with a deuterated triglycine sulfate (DTGS) detector four days after hydrogel preparation. Hydrogel samples were deposited on disposable KBr IR cards (Sigma-Aldrich, Rehovot, Israel) and dried under a vacuum. Measurements were taken using 4 cm^−1^ resolution and by averaging 2000 scans. The absorbance maxima values were determined using the OMNIC analysis program (Nicolet). The obtained transmittance spectra were smoothed by applying the Savitzky–Golay function to eliminate noise and operating the second derivative transformation on the spectra using the Peakfit software version 4.12 (SYSTAT Software Inc., Richmond, CA, USA).

#### 2.2.6. Swelling

The 1 mL hydrogel samples (three of each concentration) were prepared and placed in 35 mm plates. The initial weight (Wi) was recorded and the hydrogels were immersed in 5 mL ddH_2_O. All samples were allowed to swell for 24 h to achieve swelling equilibrium. The equilibrated swollen mass (Ws) was recorded at four different time points over 2 weeks after removing surface water from each sample by blotting with soft paper. The hydrogel samples were subsequently lyophilized, and their dry weight (Wd) was measured. The equilibrated swelling ratio (Q) was defined as the ratio of Ws to Wd. The swelling ratio of the initial state of hydrogels can be calculated from the hydrogel concentration used, e.g., 0.5%, which means 5 mg dry weight (Wd) in a total volume of 1000 μL solution or 1000 mg swelling weight (Ws). Dividing Ws by Wd resulted in a swelling ratio of 200 [21].

#### 2.2.7. Curcumin Release

A curcumin stock solution was prepared by dissolving curcumin powder in DMSO at a 10 mg/mL concentration. Curcumin stock solutions in the hydrogels were fixed to 0.05 wt % for all hydrogels. Curcumin stock solution was added to the double-distilled water before the addition of PS and/or FmocFF. The release of curcumin from the hydrogels was determined in simulated body fluid (SBF). The hydrogels were immersed in 5 mL SBF at 37 °C on an orbital shaker at 100 rpm. Aliquots of SBF were taken at predetermined time points, 100 µL of the SBF supernatants were placed in a black opaque 96-well microplate. The curcumin released from the hydrogels was measured using a Spark microplate reader (Tecan Trading AG, Switzerland) [21]. Emission at 520 nm was recorded with an excitation wavelength of 420 nm. Released curcumin concentrations were determined using a standard curve constructed by recording the absorbance of serial dilutions of curcumin solution of known concentrations. Spectra were corrected by subtraction of the corresponding buffer signal. The cumulative release of curcumin was expressed and plotted over time.

#### 2.2.8. Cell Viability Tests

MC3T3-E1 preosteoblast cells were cultured in α-MEM supplemented with 10% FCS, 100 U·ml^−1^ penicillin, and 100 U·ml^−1^ streptomycin in a Petri dish at 37 °C in a humidified atmosphere incubator containing 5% CO_2_. Hydrogels were formed in a 96-well plate and washed with a culture medium several times over 2 days to ensure complete removal of excess materials and DMSO, followed by UV sterilization for 30 min. Then, cells were seeded on the prewashed hydrogels and maintained at 37 °C in a humidified atmosphere containing 5% CO_2_. Differentiating cells were supplemented with a differentiation medium containing ascorbic acid and beta-glycerophosphate every two days for 14 days. Finally, cell viability was assessed using the MTT assay 3 days after seeding for the non-differentiated cells and at 14 days following osteogenic differentiation. MTT stock solution (5 mg/mL) was prepared in phosphate buffered saline (PBS). Then, 20 µL of this solution was added to each well, followed by a 4-h incubation. A 100 µL volume of DMSO was added to extract the MTT reduced adduct (Formazan) formed in each well. The plates were placed on a shaker for 20 min to allow the complete dissolution of the precipitated formazan in DMSO. Finally, absorbance was measured using Tecan Spark plate reader at 570 nm. The background was corrected at 680 nm. The results were presented as the percentage of viable cells with respect to control cells on the same plate [22].

#### 2.2.9. Calcium Concentration Assay

FmocFF/PS hydrogels were formed in 24-well plate. The gels were washed for three days to eliminate residual FmocFF monomers and DMSO and then sterilized by UV for 30 min. A total of 50,000 MC3T3-E1 preosteoblasts in 500 µL osteogenic medium containing ascorbic acid and beta-glycerophosphate were seeded on the prewashed hydrogels. Cells were maintained in osteogenic media for 14 days with the media replaced every two days. Following 14 days of induced osteogenic differentiation, 0.5 N HCl at 4 °C was added to all wells, and the plate was placed on a shaker for 24 h. After 24 h, calcium present in the acidic supernatant was quantified using a commercially available kit (Calcium Reagent Set, Pointe Scientific) following the manufacturer’s instructions. The absorbance of the acidic supernatant solution upon adding a calcium reagent was measured at 570 nm. A standard absorbance curve determined calcium content against a known concentration of calcium in the acidic supernatant solution. Results were normalized over the hydrogel surface area.

#### 2.2.10. Alizarin Red Mineralization Assay

Osteogenic differentiation of MC3T3-E1 preosteoblasts on the FmocFF/PS hydrogel was evaluated by Alizarin red staining. FmocFF/PS hydrogels were formed in a 96-well plate and repeatedly washed with a culture medium for three days, followed by UV sterilization for 30 min. Moreover, 10,000 MC3T3-E1 preosteoblasts per 100 µL were seeded on the prewashed hydrogels and incubated at 37 °C in a humidified atmosphere under 5% CO_2_. After two days, the cells were supplemented with osteogenic media containing ascorbic acid and beta-glycerophosphate. The cells were maintained in osteogenic media for 14 days, with the media replaced every two days. After 14 days, the cells were stained with the calcium staining dye Alizarin red. After washing off the excess dye, optical light microscopy images were acquired. Calcium deposits could be identified by their red color under a light microscope.

#### 2.2.11. Statistical Analysis

Statistical analyses were carried out by one-way ANOVA followed by Tukey’s and Sidak post hoc test (GraphPad Prism 9, CA, USA). Values were expressed as means ± SD; *p*-values < 0.05 were considered significant.

## 3. Results

### 3.1. Preparation and Structural Characterization of the Composite FmocFF/PS Hydrogels

**Figure 1:**
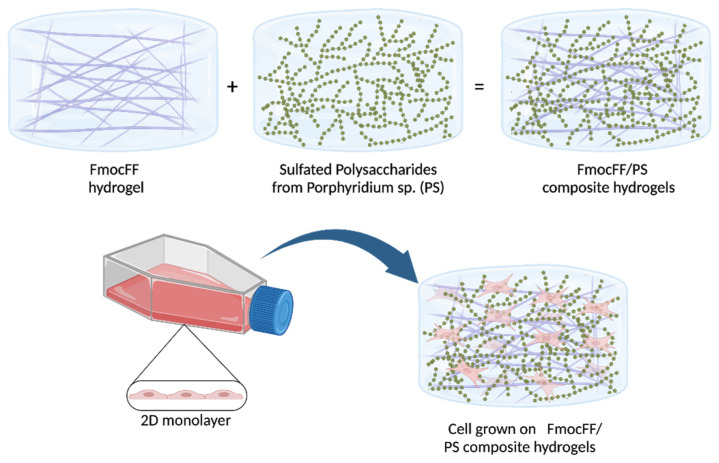
Schematic illustration of the hydrogel formation and its application as a scaffold for tissue engineering. FmocFF-peptide and sulfated polysaccharide formation into composite hydrogels, demonstrating the peptide fibrils entangles in the polysaccharide and the 3D- self-supporting hydrogel. The hydrogel potential as a scaffold for cell culture in tissue engineering is presented.

**Figure 2:**
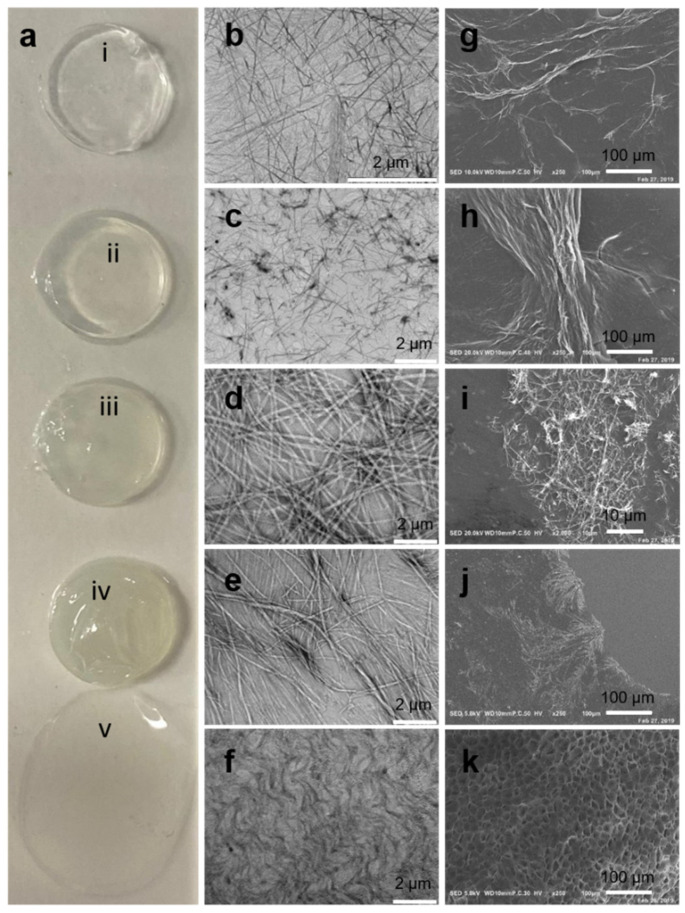
Characterization of the FmocFF/PS hydrogels. (**a**) FmocFF/PS hydrogels and the pure components FmocFF and PS. From top to bottom: (**i**) FmocFF, (**ii**) FmocFF/PS 5 mg/mL, (**iii**) FmocFF/PS 10 mg/mL, (**iv**) FmocFF/PS 20 mg/mL, (**v**) PS. (**b**–**f**) SEM images of (**b**) FmocFF, (**c**) FmocFF/PS 5 mg/mL, (**d**) FmocFF/PS 10 mg/mL, (**e**) FmocFF/PS 20 mg/mL, and (**f**) pure PS 10 mg/mL. (**g**–**j**) TEM images of (**g**) FmocFF, (**h**) FmocFF/PS 5 mg/mL, (**i**) FmocFF/PS 10 mg/mL, and (**j**) FmocFF/PS 20 mg/mL. (**k**) SEM of the lyophilized pure PS 10 mg/mL.

FmocFF peptide (100 mg/mL) was mixed with PS (10 mg/mL) and placed in 9 mm silicon mold to form composite hydrogels (Figure 1) at a ratio of 1:1 at three concentrations (5 mg/mL, 10 mg/mL, and 20 mg/mL). All three concentrations gave rise to a self-supported, homogenous hydrogel formed within a few minutes, similar to the pure FmocFF hydrogel (Figure 2a), while PS alone did not form a self-supporting hydrogel. As the concentration increased, the composite hydrogels appeared more opaque (Figure 2a).

TEM and SEM analyses demonstrated the nanofibrillar architecture of the composite hydrogels (Figure 2c–e, h–j, respectively), similar to the nanofibrous architecture of FmocFF alone (Figure 2b,g).

**Figure 3:**
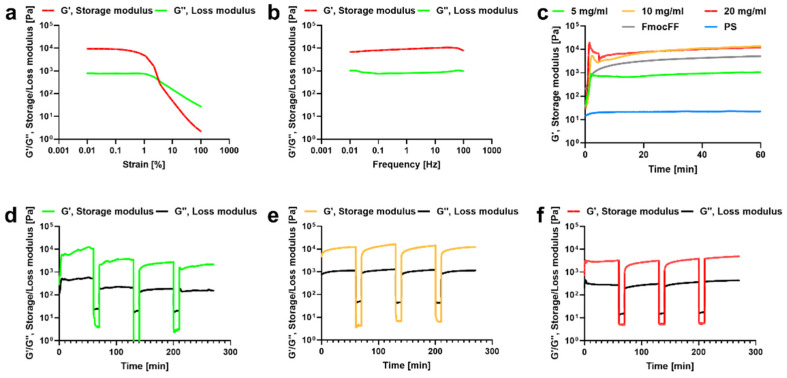
Rheology of the FmocFF/PS hydrogels. (**a**) Strain sweep of the 10 mg/mL FmocFF/PS composite hydrogel. (**b**) Frequency sweep of the 10 mg/mL FmocFF/PS composite hydrogel. (**c**) In situ time sweep oscillation measurements of the FmocFF, PS, and composite FmocFF/PS hydrogels. (**d**) Time sweep of the 5 mg/mL FmocFF/PS composite hydrogel under alternate step strain switched from 0.1% to 100%. (**e**) Time sweep of the 10 mg/mL FmocFF/PS composite hydrogel under alternate step strain switched from 0.1% to 100%. (**f**) Time sweep of the 20 mg/mL FmocFF/PS composite hydrogel under alternate step strain switched from 0.1% to 100%.

Next, we aimed to determine the physical properties of the different composite hydrogels. Storage modulus (G’) and loss modulus (G”) of the hydrogels were measured by strain sweep analysis (at 5 Hz frequency). To study the oscillatory strain, the hydrogels were subjected to 0.01–100% strain sweep at a constant frequency, resulting in a broad linear viscoelastic region of up to 5% strain (Figure 3a). Frequency sweep experiments at a constant strain using a frequency range of 0.1–100 Hz also showed a broad LVR (Figure 3b). We determined LVR based on both the dynamic strain sweep and frequency sweep tests and conducted a time sweep measurement at a fixed strain of 0.1% and frequency of 5 Hz. The frequency sweep results suggest that G’ and G” were independent of the applied frequency, showing a predominantly elastic-like behavior (G’) relative to the viscous component (G”). Moreover, the ratio between G’ and G” was <1, indicating the viscoelastic profile of the hydrogels.

Time sweeps analysis demonstrated that the pristine PS has a low G’ value of 22 Pa (Figure 3c). In contrast, FmocFF peptide hydrogels showed a G’ value of 10,216 Pa. The 5 mg/mL FmocFF/PS composite hydrogel demonstrated an intermediate G’ value of 1230 Pa. The 10 mg/mL and 20 mg/mL FmocFF/PS composites showed higher G’ values compared to the pure FmocFF (13,698 Pa and 11,930 Pa, respectively).

To examine the self-healing nature of the hydrogels, an alternate step strain experiment was conducted [29]. The hydrogels were subjected to seven cycles of time sweep experiments with low (0.1%) and high (100%) strain values (Figure 3d–f). At 100% strain, all hydrogels were converted to a solution state, as evident from the modulus values (G’ < G”). When the strain was reduced to 0.1%, the hydrogels recovered (G’ > G”). This characteristic was consistent over multiple cycles, illustrating the reproducibility of the self-healing nature. It is noteworthy that the 10 mg/mL and 20 mg/mL FmocFF/PS composite hydrogels showed better thixotropic behavior than the 5 mg/mL FmocFF/PS hydrogel.

**Figure 4:**
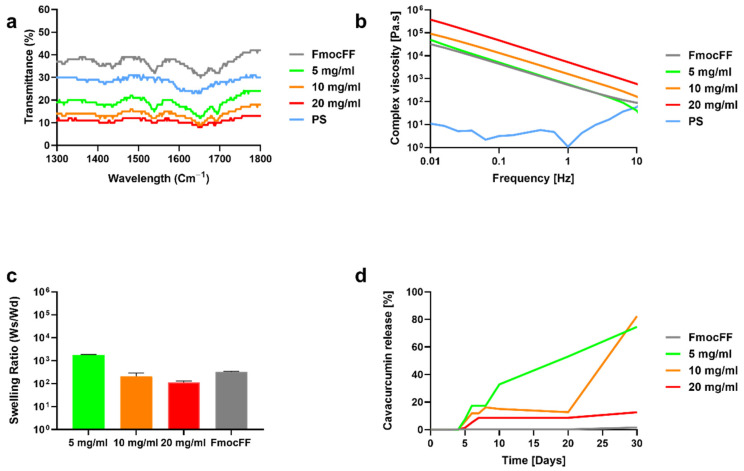
Structural and mechanical characterization of the composite FmocFF/PS hydrogels. (**a**) FTIR spectra of FmocFF/PS composites, FmocFF and PS. (**b**) The complex viscosity (η”) versus frequency plots of the pure and composite samples. (**c**) Swelling ratio of the FmocFF/PS composite hydrogels and FmocFF. Data analyzed using one-way ANOVA. (**d**) Curcumin release profile from FmocFF and the composite FmocFF/PS hydrogels.

FTIR spectra were measured for the FmocFF/PS composite hydrogels and the individual components (Figure 4a). The distinct peaks observed at 1647 cm^−1^ and at 1694 cm^−1^ were observed for all the composite hydrogels as well as the pristine peptide hydrogel. These peaks are associated with amide I C=O stretching vibration and are directly related to the backbone conformation [30]. The two peaks observed at 1647 cm^−1^ and at 1694 cm^−1^ in the composite hydrogels suggest the presence of a carbamate moiety and indicate that the composite hydrogels are rich in β-sheets, similar to the FmocFF hydrogel. The FTIR spectra of the PS showed a band at 1600 cm^−1^ corresponding to a carboxylic ester group.

Figure 3b shows the complex viscosity variation with respect to the frequency for the FmocFF/PS composite hydrogels and the pure components. At a low frequency, high viscosity values of the composite hydrogels decreasing with the increase in frequency were observed. Higher viscosity rates were found at higher concentrations of the composite. We further studied the relationship between the concentration of the composites and their swelling. The 5 mg/mL hybrid hydrogel showed high swelling ratio of 1800 Ws/Wd, however, the other hydrogels showed lower swelling ratio of 210 Ws/Wd and 111 Ws/Wd for the 10 mg/mL and 20 mg/mL hybrid hydrogels, respectively (Figure 4c). The swelling ratio was significantly higher in the 5 mg/mL hybrid hydrogel when compared to the 10 mg/mL and 20 mg/mL hybrid hydrogels (*p* < 0.0001). However, no difference was found between the swelling ratios of the 10 mg/mL and 20 mg/mL hybrid hydrogels (*p* = 0.967).

### 3.2. Curcumin Release from the FmocFF/PS Composite Hydrogels

The hydrogels were further assessed as vehicles for bioactive molecules using encapsulated curcumin, a naturally fluorescent molecule due to its polyphenolic structure. The composite hydrogels at the three concentrations maintained their self-supported nature in the presence of curcumin (data not shown). Fluorescence spectroscopy analysis clearly showed that curcumin release was slower when the concentration of the hydrogel was higher (Figure 4d). In the pure FmocFF and the 20 mg/mL FmocFF/PS composite hydrogel, curcumin release started after four and five days, respectively, and a slow-release pattern was observed. After 30 days, only 15% of the curcumin was released from the composite hydrogel. Similarly, in the 5 mg/mL and 10 mg/mL FmocFF/PS hydrogels, curcumin release began after five days. However, a sharp increase in curcumin release was observed for the 5 mg/mL and 10 mg/mL FmocFF/PS hydrogels at day 10 and at day 20, respectively. In these two samples, after 30 days, approximately 80% of the curcumin was released (Figure 4d).

### 3.3. Biocompatibility of the FmocFF/PS Composite Hydrogels

**Figure 5:**
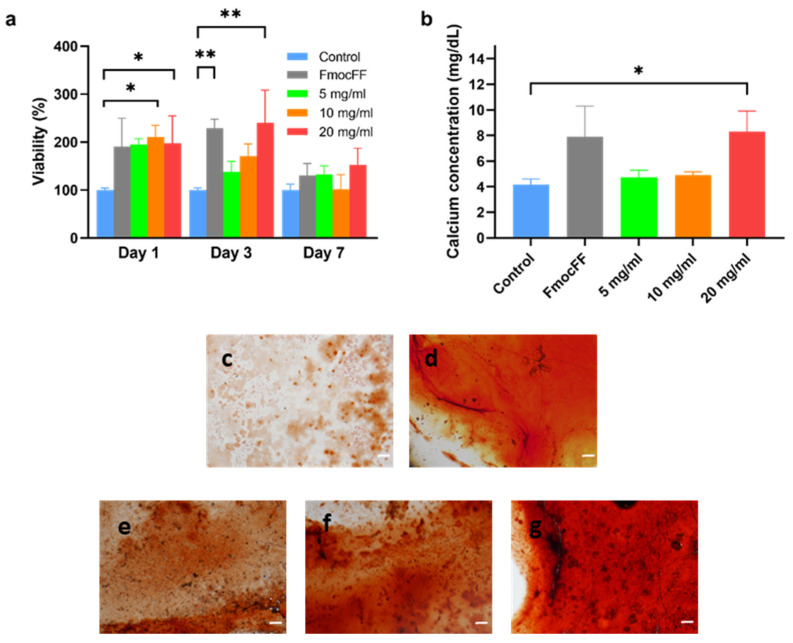
Biocompatibility of the composite FmocFF/PS hydrogels and osteogenic differentiation. (**a**) Quantitative measurements of MC3T3-E1 preosteoblasts viability one, three, and seven days post-seeding on the hydrogel compared to MC3T3-E1 preosteoblasts grown in media, without the hydrogel, used as a negative control (Control). Data analyzed using one-way ANOVA. * *p* < 0.05, ** *p* < 0.01. (**b**) Quantification of the calcium content in the supernatant of the FmocFF/PS hydrogels compared to the control 14 days after seeding in osteogenic media. Data analyzed using one-way ANOVA. * *p* < 0.05. (**c**–**f**) Optical microscope images of MC3T3-E1 preosteoblasts stained with Alizarin red following 14 days of osteogenic differentiation (scale bar=100 µm). (**c**) After seeding on tissue culture plastic (control). (**d**) After seeding on pure FmocFF hydrogel. (**e**) After seeding on 5 mg/mL FmocFF/PS hydrogel. (**f**) After seeding on 10 mg/mL FmocFF/PS hydrogel. (**g**) After seeding on 20 mg/mL FmocFF/PS hydrogel.

To test the applicability of the composite FmocFF/PS hydrogels as scaffolds for tissue engineering in vitro, MC3T3-E1 preosteoblasts were seeded on the hydrogels and quantified one, three, and seven days post-seeding compared to cells seeded directly on the plate as a control. Figure 5a shows that the composite hydrogels supported cell viability and proliferation for up to seven days. Higher viability was observed on the 10 mg/mL and 20 mg/mL FmocFF/PS composite hydrogels compared to the control at all time points, however this difference was statistically significant only after 1 day for the 10 mg/mL hydrogel (*p* = 0.0224) and after 1 and 3 days for the 20 mg/mL hydrogel (*p* = 0.0453 and *p* = 0.0022, respectively).

### 3.4. Osteodifferentiation of MC3T3-E1 Preosteoblasts on FmocFF/PS Composite Hydrogels

The 20 mg/mL FmocFF/PS hydrogel induced significantly higher calcium deposition in MC3T3-E1 preosteoblasts grown in osteogenic media compared to cells grown on tissue culture plastic (*p* = 0.0360) (Figure 5b). This result was confirmed by the Alizarin red staining (Figure 5c–g). The staining intensity was higher as the hydrogel concentration increased (Figure 5e–g).

## 4. Discussion

In the present study, the sulfated polysaccharide derived from the red marine microalga *Porphyridium* sp. was physically modified by the incorporation of FmocFF, an extra short aromatic self-assembling peptide to form a polysaccharide-based interpenetrating polymer network (IPN) hydrogel [31]. The mixture of the peptide and the polysaccharide at three different concentrations led to the formation of biomimetic, injectable, stable hydrogels with enhanced G’ values compared to the hydrogels’ pure components. This observation is in accordance with previously reported IPNs [32].

The FmocFF/PS hydrogels’ nanofibrous appearance resembles the architecture of the natural ECM. Thus, these matrices may allow nutrient transmission and potentially provide mechanical integrity as 3D environments for cell attachment and tissue regeneration [33]. Furthermore, the broad range of G’ values may indicate the ability of the composite hydrogels to serve as potential scaffolds in tissue engineering that allow osteogenic, chondrogenic and adipogenic differentiation [34,35,36].

FmocFF is hypothesized to be the driving force of the supramolecular organization with the PS, producing a more rigid hydrogel compared to the individual components. On the other hand, the higher viscosity rates found at higher concentrations of the hydrogel may stem from the entangled networks of polymer chains and fibrils [37]. The shear-thinning and thixotropic behavior of the composite hydrogels exemplifies their self-healing nature and injectability that makes them suitable candidates for 3D-printed tissue engineering scaffolds under physical conditions [38,39].

Biomaterials capable of controlled delivery of biomolecules are desirable in tissue engineering. In this work, the composite hydrogels exhibited the capability of effective delivery of curcumin. Furthermore, the rate of curcumin release was regulated by the hydrogel concentration. Finally, the composite hydrogel exhibiting the highest concentration enabled significant calcium deposition by differentiating MC3T3-E1 preosteoblasts, suggesting its osteoconductive properties. This observation may be ascribed to the high stiffness of this hydrogel [35,36].

There are several limitations of this study. Most notably, MC3T3-E1 preosteoblasts were used for the viability and osteodifferentiation assays. Although this is one of the most commonly used osteoblast-like cell lines for evaluating osteoinductive characteristics of biomaterials, still, the use of bone marrow-derived mesenchymal stem cells would have better represented the conditions in vivo. Additionally, the hydrogels are novel and require further in vivo studies to assess their biocompatibility as well as their ability to induce tissue regeneration.

## 5. Conclusions

This work demonstrated the enhancement of the mechanical properties of the PS derived from marine algae by incorporating the FmocFF peptide. Composite hydrogels at three concentrations were obtained with an ECM-mimicking nanofibrous architecture. All hydrogels were stable, biocompatible, and allowed calcium deposition after incubation with MC3T3-E1 preosteoblasts. Furthermore, they allowed the sustained release of curcumin, controlled by changing the hydrogel concentration. Due to the tunable mechanical properties, biocompatibility, and release of bioactive molecules, these hydrogels may serve as tissue engineering scaffolds that potentially induce different cell lineage differentiation.

## Data Availability

Data is contained within the article.
